# The VISTA/VSIG3/PSGL-1 axis: crosstalk between immune effector cells and cancer cells in invasive ductal breast carcinoma

**DOI:** 10.1007/s00262-024-03701-w

**Published:** 2024-06-04

**Authors:** Mateusz Olbromski, Monika Mrozowska, Aleksandra Piotrowska, Beata Smolarz, Hanna Romanowicz

**Affiliations:** 1 Department of Histology and Embryology, Department of Human Morphology and Embryology, Wroclaw Medical University, Chalubinskiego 6a, 50-368 Wroclaw, Poland; 2 Department of Pathology, Polish Mother’s Memorial Hospital Research Institute, 93-338 Lodz, Poland

**Keywords:** Breast cancer, VISTA, VSIG3/IGSF11, PSGL-1, CD45, CD68

## Abstract

A checkpoint protein called the V-domain Ig suppressor of T cell activation (VISTA) is important for controlling immune responses. Immune cells that interact with VISTA have molecules, or receptors, known as VISTA receptors. Immune system activity can be modified by the interaction between VISTA and its receptors. Since targeting VISTA or its receptors may be beneficial in certain conditions, VISTA has been studied in relation to immunotherapy for cancer and autoimmune illnesses. The purpose of this study was to examine the expression levels and interactions between VISTA and its receptors, VSIG3 and PSGL-1, in breast cancer tissues. IHC analysis revealed higher levels of proteins within the VISTA/VSIG3/PSGL-1 axis in cancer tissues than in the reference samples (mastopathies). VISTA was found in breast cancer cells and intratumoral immune cells, with membranous and cytoplasmic staining patterns. VISTA was also linked with pathological grade and VSIG3 and PSGL-1 levels. Furthermore, we discovered that the knockdown of one axis member boosted the expression of the other partners. This highlights the significance of VISTA/VSIG3/PSGL-1 in tumor stroma and microenvironment remodeling. Our findings indicate the importance of the VISTA/VSIG3/PSGL-1 axis in the molecular biology of cancer cells and the immune microenvironment.

## Introduction

Breast cancer is one of the most common types of cancer worldwide. In 2020, an estimated 2.3 million new cases of breast cancer were diagnosed globally [1–3]. It is considered to be one of the main causes of cancer morbidity and mortality in women. Early detection, education, and advances in treatment have contributed to improved survival rates and outcomes for breast cancer patients. However, ongoing research and efforts to promote early detection and access to quality care remain important to address this serious health issue 

The search for new cancer markers is a crucial element of cancer research, mostly because biomarkers may be identified in the blood, tissues, or other physiological fluids. Therefore, they can aid in the early detection of cancer, even before the appearance of symptoms. Early identification is frequently associated with better treatment results and greater survival rates [5, 10–15].

Targeting the VISTA protein, a V-domain Ig suppressor of T cell activation, is becoming increasingly important in cancer research because it has been proven to be an important immunological checkpoint and therapeutic target [16, 17]. A significant number of immune cells express VISTA, which is associated with the control of immunity by controlling the immune response, including inhibition of T cell activation [16–22]. Recent research findings highlight some of the key aspects of VISTA's involvement in immune regulation and its potential as a target for cancer therapy. The V-set and Immunoglobulin Domain-Containing Protein 3 (VSIG3) and the glycoprotein ligand P-selectin 1 (PSGL-1) bind VISTA, and the signal may be two-way [23–25]. At acidic pH, as in the tumor microenvironment (TME), VISTA binds to PSGL-1, but not at physiological pH.

VSIG3 is a member of the Ig superfamily expressed on a variety of non-hematopoietic cells, but its immunosuppressive activity has only recently been described [23–25]. The exact mechanism by which VSIG3 signaling suppresses tumor-associated macrophages (TAMs) and tumor-infiltrating lymphocyte (TIL)-mediated responses remains unknown. Human VSIG3 is expressed in tumor cells, including gastric cancer cells and hepatocyte cells [29–32]. Although VSIG3 knockdown suppresses tumor growth in St-4 gastric cancer cells in vitro, the immunological role of VSIG3 in tumors in vivo has not been demonstrated [33]. Both VSIG3 and PSGL-1 interact with overlapping but distinct regions of VISTA, as shown by structural and mutational analyses.

P-selectin glycoprotein ligand-1 is associated with the immune system and cell adhesion. It is a significant factor in the recruitment and inflammation of leukocytes [34–37]. PSGL-1, a protein found on activated endothelial cells, is found on the surface of white blood cells (leukocytes) and interacts with P-selectin. 

In conditions such as wound healing and tumors, where the pH can be 5.85–6.5, the role of PSGL-1 as an immune checkpoint has been clarified. Therefore, it is plausible that human PSGL-1 is a binding partner of human VISTA in tumors at a low pH [21, 25]. The role of PSGL-1 in antitumor immunity is still unknown, and there are limited data directly addressing its role. Although VISTA and PSGL-1 may function as both ligands and receptors, it remains to be determined whether the VISTA/PSGL-1 interaction occurs exclusively in trans or can also act in cis to inhibit activation [21, 38, 39]. The immunological activity of PSGL-1 is currently being reassessed, making PSGL-1 itself a potential therapeutic target.

VISTA activity causes myeloid and naïve mammalian T cells to become dormant while suppressing T cell activation and cytokine production. It may increase peripheral tolerance by increasing activation-induced T cell apoptosis [21, 22, 41, 42]. VISTA is preferentially upregulated in myeloid-derived suppressor cells (MDSCs) in response to hypoxia and may contribute to the immunosuppressive role of myeloid cells by reducing Toll-like receptor (TLR) signaling, inhibiting cell migration, and inducing myeloid reprogramming toward decreased myeloid cells [25, 43–46]. Furthermore, there may be a correlation between the overexpression of VISTA and the increased synthesis of the pro-inflammatory cytokines interleukin IL-6, tumor necrosis factor (TNF)-α, and IL-12, as well as increased production of IL-10 and other anti-inflammatory mediators [22, 47–50].

Antibodies against VISTA are being tested in clinical trials for the treatment of numerous malignancies; acid-targeting medicines in the TME can diminish the immunosuppressive action of acid tea and work well with VISTA therapy or checkpoint inhibition. Recent observations have shown that higher VISTA expression is related to better clinical results, whereas PD-L1 expression is associated with worse outcomes in patients with malignant cell carcinoma [17]. VISTA is mostly expressed on TME immune cells in most human malignancies and mouse models, while it has also been found in lung, kidney, and colorectal tumor cells, endometrial tissue, and human ovaries [19, 20, 43, 47, 51–58]. Tumor immunity is impacted by VISTA in both positive and negative ways. In a variety of cancer forms, VISTA functions as an inhibitory immunological checkpoint in addition to potentially serving as a stimulatory immune checkpoint. Since the precise process is yet unknown, it is essential to first determine VISTA's and its partners' potential as cancer diagnostic and predictive tools and to thoroughly define their mechanisms of action. Additionally, upregulation of VISTA in immune cells following immune checkpoint inhibition has been demonstrated in multiple studies [25, 59–63]. In light of this and the significance of VISTA expression on tumor and stromal cells, it seems that VISTA represents a promising target for therapy.

Therefore, this study aimed to evaluate the expression of VISTA and other immune checkpoint molecules in human breast cancer. Understanding the role of immune checkpoint molecules in cancer, including the proteins within the VISTA/VSIG3/PSGL-1 axis, may be crucial for developing effective immunotherapies and prognostic markers. Investigating whether VISTA’s axis expression levels are prognostic in breast cancer patients could provide valuable insights into disease progression and potential therapeutic strategies.

## Materials and methods

### Patients and clinical samples

The experiments were conducted using archival paraffin blocks of invasive ductal breast carcinomas (n = 284) and fibrocystic breast disease (FBD) mastopathy (n = 27) obtained during surgical resection at the Polish Mother's Memorial Hospital Research Institute in Lodz between 2010 and 2016. Paraffin slices of the malignant tissues were stained with hematoxylin and eosin (H&E) to ensure that immunohistochemistry (IHC) analyses were correct. Table 1 summarizes the clinical data obtained from the hospital archives.

**Table 1:** Clinicopathological data of the studied cases of invasive ductal breast carcinoma

Characteristic	No. (%) of patients (*n* = 284)
*Tumor size*	
T1 (< 2 cm)	89 (31.34)
T2 (2–5 cm)	75 (26.41)
T3 (> 5 cm)	68 (23.94)
T4	52 (18.31)
*Tumor stage—pT*	
1	76 (26.76)
2	145 (51.06)
3	36 (12.68)
4	27 (9.51)
*Grading*	
I	75 (26.41)
II	144 (50.70)
III	65 (22.89)
*Nodal status pN*	
N0	131 (46.13)
N1	83 (29.23)
N2	39 (13.73)
N3	31 (10.92)
*ER status*	
Positive	182 (64.08)
Negative	102 (35.92)
*PR status*	
Positive	189 (65.55)
Negative	98 (33.45)
*HER2 status*	
Positive	174 (61.72)
Negative	110 (38.73)
*Triple negative status*	
Positive	36 (12.68)
Negative	248 (87.32)

The immunocytochemical expression of VISTA, VSIG3, PSGL-1, CD45, and CD68 was investigated using the ImmunoReactive Score (IRS) developed by Remmele and Stenger, which considers both the intensity of the reaction color (staining) and the proportion of positively stained cells (Table 2).

**Table 2:** Evaluation of immunohistochemical reactions with the use of the ImmunoReactive score (IRS)

% Positive cells	Intensity of reactions	IRS
0 = no positive cells	0 = no color reaction	0–1 = negative
1 ≤ 10% positive cells	1 = mild reaction	2–3 = mild
2 = 10–50% positive cells	2 = moderate reaction	4–8 = moderate
3 = 51–80% positive cells	3 = strong reaction	9–12 = strong
4 ≥ 81% positive cells		

The final score ranged from 0 to 12. The Mann–Whitney U-test, the ANOVA Kruskal–Wallis test, and the Spearman’s test were used for statistical analysis. When p0.05, differences were considered statistically significant.

### Cell lines

Breast carcinoma cell lines MCF-7, T-47D, MDA-MB-231 (obtained from the American Type Culture Collection ATCC, Manassas, VA, USA), SK-BR-3, BT-474 (from the Cell Lines Collection of the Ludwik Hirszfeld Institute of Immunology and Experimental Therapy of the Polish Academy of Science, Wroclaw, Poland), and MDA-MB-231/BO2 (courtesy of Dr. Philippe Clezardin), as well as ME16C normal breast epithelial cells (American Type Culture Collection ATCC), were used in our study. Breast cancer cell lines were grown in α-MEM supplemented with 10% fetal calf serum (FCS; Invitrogen Carlsbad, CA, USA), antibiotics, and 2 mM l-glutamine (Lonza, Basel, Switzerland). MEGM Bulletkit medium (Lonza, Basel, Switzerland) was used to cultivate Me16C cells. FBS (Sigma) was added to all the media at a final concentration of 10%. The cell lines were grown in 5% CO_2_ at 37 °C. The aggressiveness potential of each of the investigated cell lines was ranked from the lowest to the highest.

### Immunohistochemistry (IHC)

Cancer and non-cancerous tissue samples were fixed in 10% buffered formalin and embedded in paraffin for immunohistochemistry (IHC) assays. Mouse polyclonal antibodies against VISTA (1:500; PA5-113459, Thermo Fisher Scientific), mouse monoclonal antibodies against VSIG3 (1:400, MA5-26624, Thermo Fisher Scientific), rabbit polyclonal antibodies against PSGL-1 (1:500, PA5-140244, Thermo Fisher Scientific), anti-CD45 mouse antibody cocktail (1:200, MA5-13197, Thermo Fisher Scientific), and anti-CD68 mouse monoclonal antibodies (1:200, 14-0688-82, Thermo Fisher Scientific) were used for the immunohistochemical examination of the studied markers. IHC was performed using Autostainer Link 48 (DakoCytomation, Glostrup, Denmark) to provide consistent and repeatable results.

### RNA extraction, cDNA synthesis, and real-time PCR reactions

Total RNA was extracted using the RNeasy Mini Kit (Qiagen, Hilden, Germany) and transcribed into cDNA using the iScript cDNA Synthesis Kit (Bio-Rad Laboratories, Hercules, CA, USA), according to the manufacturer’s protocol. RT-qPCR was performed in 20 l volumes using a 7500 Real-time PCR System with iTaq Universal Probes Supermix (Bio-Rad Laboratories, Hercules, CA, USA). Applied Biosystems also provided the following TaqMan-specific probes used in the experiment: Hs00735289_m1 for *C10orf54* (VISTA), Hs00541322_m1 for *IGSF11* (VSIG3), Hs00356602_m1 for *SELPIG* (PSGL-1), and Hs99999903_m1 for *ACTB* as a reference gene. All reactions were performed in triplicate under the following conditions: polymerase activation at 50 °C for 2 min, initial denaturation at 94 °C for 10 min, followed by 40 cycles of denaturation at 94 °C for 15 s, and annealing and elongation at 60 °C for 1 min. The Ct method was used to determine the relative mRNA expression of the markers.

### SDS-PAGE and western blotting

Cell lines were lysed on ice in Cell Lysis Buffer (Thermo Fisher Scientific, Waltham, MA, USA) containing a cocktail of inhibitors (Sigma, St. Louis, MO, USA), 250 units of Benzonase® (Merck Millipore, Bedford, MA, USA), and 2 mM phenylmethanesulfonyl fluoride (PMSF). Lysates containing 30 µg of total protein were combined with 4 × SDS-PAGE gel loading buffer (200 mM Tris–HCl (pH 6.8), 400 mM DTT, 8% SDS, 0.4% bromophenol blue, 40% glycerol), loaded on 10% acrylamide gels, and separated by SDS-PAGE under reducing conditions before being transferred to PVDF membranes. Following protein transfer, the membranes were incubated for 1 h at room temperature in blocking solution (4% BSA in TBST buffer), followed by overnight incubation at 4 °C with anti-VISTA (1:500), anti-VSIG3 (1:400), and anti-PSGL-1 (1:500) antibodies. The membranes were then washed with TBST buffer and incubated for 1h at room temperature with HRP-conjugated anti-rabbit and anti-mouse secondary antibodies, diluted 1:3000 (709-035-149 and 715035-150, respectively; Jackson ImmunoResearch, Mill Valley, CA, USA), and washed and treated with the Immun-Star HRP Chemiluminescence Kit (Bio-Rad). Rabbit anti-human β-actin monoclonal antibody (#4970; Cell Signaling Technology, Danvers, MA, USA), diluted 1:1000, was used as the internal control. Western blotting results were analyzed using the ChemiDoc MP system (Bio-Rad).

### siRNA transfection

Ambion pre-designed siRNAs, including GAPDH siRNA as a positive control and scrambled sequence siRNA as a negative control, were used in the experiments. The specific siRNAs used were as follows: s34467 for *C10orf54*, s12688 for *SELPIG*, and s45689 for *IGSF11*. MDA-MB-231 and T-47D cells were grown in 6-well plates, as previously described. The concentration of siRNA and quantity of transfection reagent were determined experimentally. Cells were trypsinized, centrifuged at 1000 rpm for 5 min at 4 °C, and resuspended in new media before transfection. Ambion's siPORT NeoFX (6µl/well) lipid-based transfection reagent and siRNAs (50 nM final concentration) were individually diluted in OptiMEM and mixed together. After 10 min, the transfection complexes were overlaid with 2 × 10^5^ cells/well. VISTA/VSIG3/PSGL-1 silencing was verified.

### Statistical analysis

The Shapiro–Wilk test was performed to assess the normality assumption of the groups investigated. The Wilcoxon signed-rank test was used to examine differences between the LSCC and NMLT groups. In addition, the Spearman correlation test was used to analyze the existing relationships. Prism 8.1.0 (GraphPad Software, La Jolla, CA, USA) was used for all statistical analyses. Results were considered statistically significant at p < 0.05.

## Results

### VISTA/VISG3/PSGL-1 axis is associated with breast tumor features

**Fig. 1:**
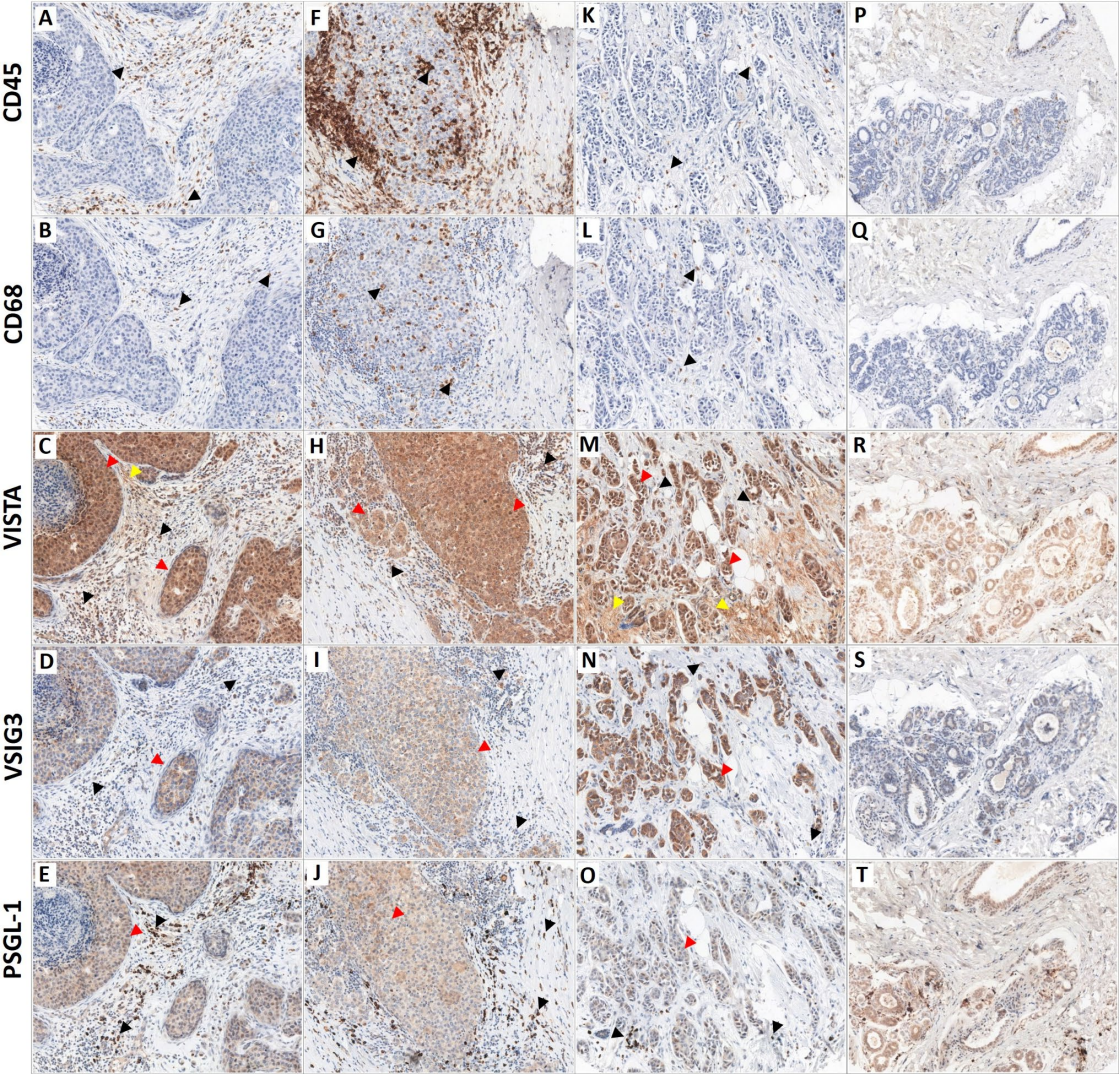
Serial sectioning slides illustrating the expression of CD45, CD68, VISTA, VSIG3, and PSGL-1 in breast cancer tissues (**A–O**) and mastopathies (**P–T**). Tumor-infiltrating lymphocytes (TIL—CD45-positive cells) and tumor-associated macrophages (TAM—CD68-positive cells), which are classified as tumor-infiltrating immune cells (TIICs), were found to express CD45 and CD68 (black arrows). Cancer cells that express the VISTA/VSIG-3/PSGL-1 proteins are indicated by red arrows. Yellow arrows are used to represent VISTA stromal expression. The initial magnification was × 400

IHC analysis of the BC and FBD samples revealed that the expression levels of all the studied proteins were significantly elevated in breast cancer cells compared to mastopathy cases (Fig. 1). Membranous expression of VISTA was noticed in 275 (97.01%) IDC samples, compared to 11 (40.74%) in the FBD samples. Moreover, VISTA expression was observed in tumor-infiltrating immune cells (TIICs) in 252 (88.81%) cases of IDC. It is worth mentioning that 27 (9.51%) cases of IDC showed a stromal expression of the VISTA protein in cancer-associated fibroblasts (CAFs), as shown in Fig. 1C and M. VSIG3 showed a membranous expression pattern in 252 (88.81%) cases of IDC and in 36 (12.69%) TIIC areas of IDC cases, compared to 8 (29.63%) cases of FBD. PSGL-1 expression was noticed in 197 (69.40%) cases of IDC and in 207 (73.13%) TIIC regions of IDC cases, compared to 6 (22.22%) cases of FBD.

**Fig. 2:**
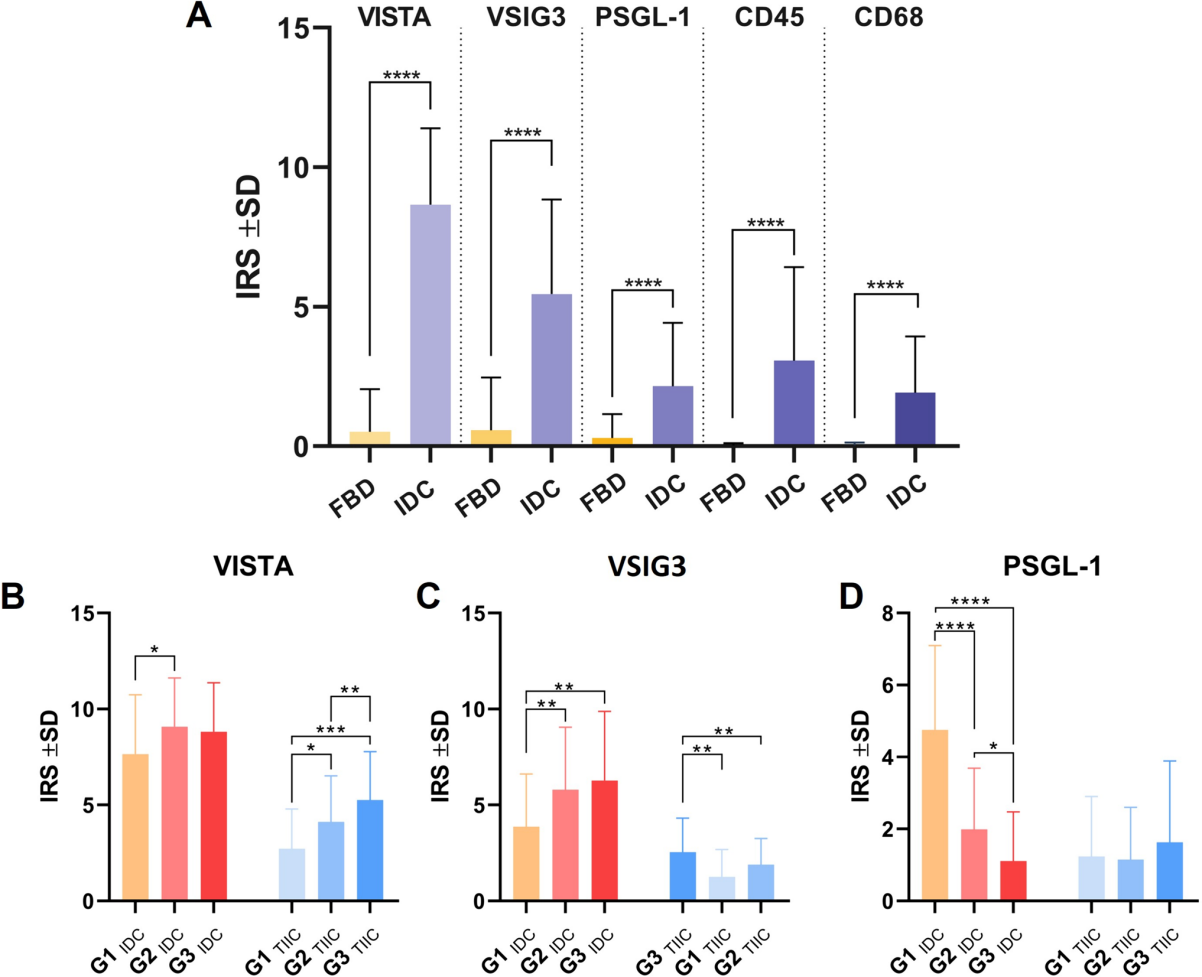
Statistical analysis of VISTA, VSIG3, PSGL-1, CD45, and CD68 proteins in FBD (n = 28) and IDC (n = 284) cases. When compared to mastopathies, all of the investigated markers have higher expression levels in BC samples (p < 0.0001; Mann–Whitney test) (**A**). Expressions of VSIG3 (**C**), PSGL-1 (**D**), and VISTA (**B**) in relation to malignancy grade G in BC (Mann–Whitney test)

Using IHC, we determined the membranous localization and expression levels of VISTA, VSIG3, PSGL-1, CD45, and CD68 patterns in 27 cases of FBD and 284 cases of IDC. These values were the following: VISTA (IRS 8.66 ± 2.74 vs. 0.90 ± 1.90, p < 0.0001; Mann–Whitney test), VSIG3 (IRS 5.46 ± 3.39 vs. 0.92 ± 2.03, p < 0.0001; Mann–Whitney test), PSGL-1 (IRS 2.15 ± 2.28 vs. 0.48 ± 1.27, p < 0.0001; Mann–Whitney test), CD45 (IRS 3.07 ± 3.36 vs. 0.02 ± 0.09, p < 0.0001; Mann–Whitney test), and CD68 (IRS 1.92 ± 2.02 vs. 0.09 ± 0.01, p < 0.0001; Mann–Whitney test) (Fig. 2A).

A statistical analysis showed that VISTA shows higher expression levels when comparing the histological grades of IDC (G1 vs. G2, p = 0.02; Mann–Whitney test) and in the TIIC regions of IDC (G1 vs. G2, p = 0.009; G2 vs. G3, p = 0.04; and G1 vs. G3, p = 0.0002; Mann–Whitney test) (Fig. 2B). VSIG3 showed a statistically higher expression in histologically graded G2 cases compared to G1 ones (G1 vs. G2, p = 0.007; Mann–Whitney test) and in G3 vs. G1 cases (G1 vs. G3, p = 0.0087; Mann–Whitney test). The same pattern was noticed when analyzing TIIC regions of IDC samples: (G1 vs. G2, p = 0.043; and G1 vs. G3, p = 0.005; Mann–Whitney test) (Fig. 2.C).

In contrast to VISTA and VSIG3 expression patterns, PSGL-1 showed a decreasing expression trend associated with histopathological grading of IDC (G1 vs. G2, p < 0.0001; G2 vs. G3, p = 0.02; and G1 vs. G3, p < 0.0001; Mann–Whitney test) (Fig. 2D).

**Fig. 3:**
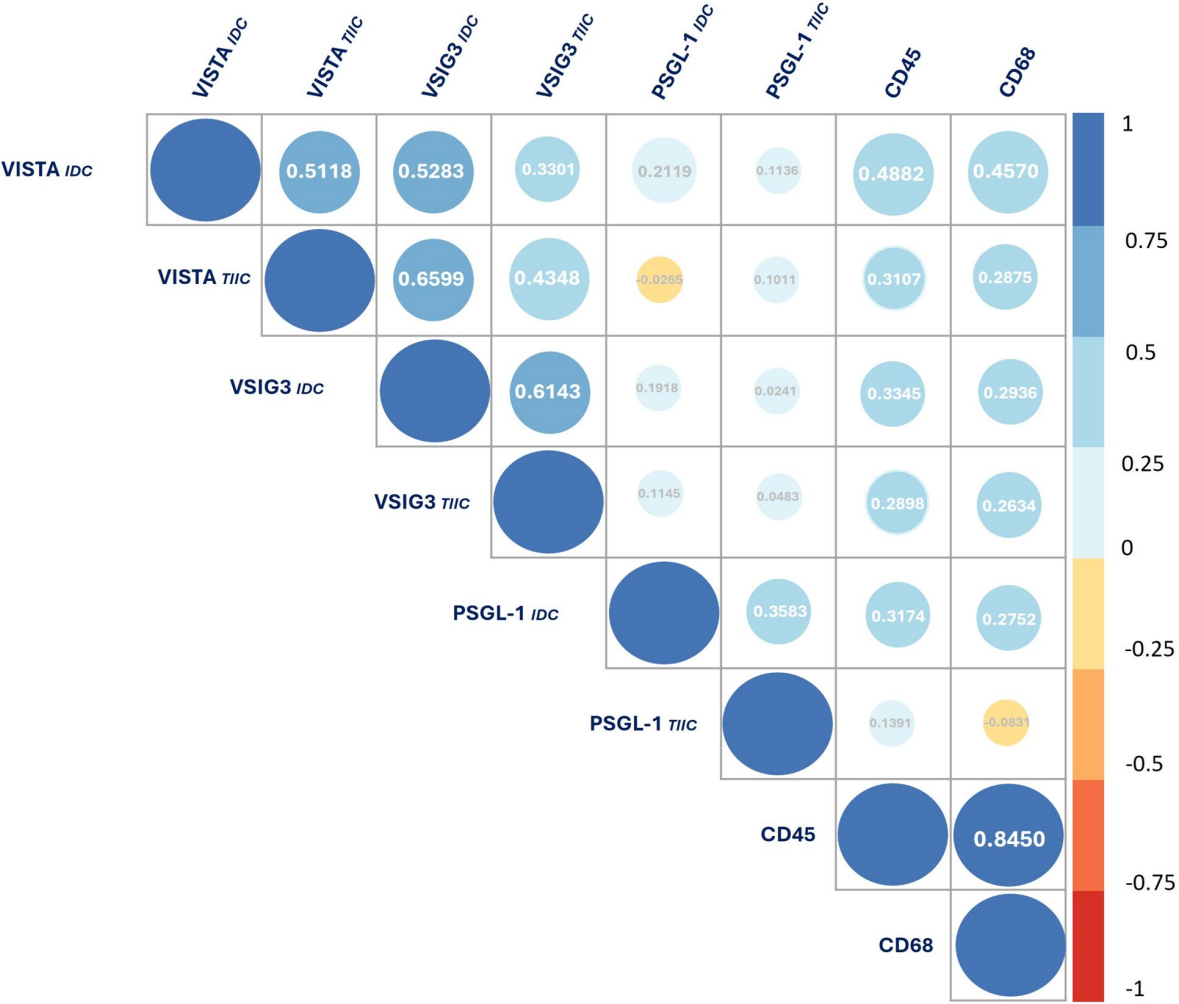
Spearman rank correlation matrix for VISTA, VSIG3, PSGL-1, CD45, and CD68 for cancer cells and TIIC populations among 284 breast cancer samples (IDC). Positive correlations are shown in blue, and negative correlations are shown in red. The circle's size and color intensity correspond to the correlation coefficients. The correlation coefficients and matching colors are displayed in the color bar on the right side of the graphic

The Spearman correlation test revealed a high positive correlation between VISTA_*IDC*_ and VISTA_*TIIC*_ (r = 0.51, p < 0.0001), VISTA_*IDC*_ and VSIG3_*IDC*_ (r = 0.53, p < 0.0001), VISTA_*IDC*_ and VSIG3_*TIIC*_ (r = 0.33, p < 0.0001), VISTA_*IDC*_ and CD45 (r = 0.49, p < 0.0001), VISTA_*IDC*_ and CD68 (r = 0.46, p < 0.0001), VISTA_*TIIC*_ and VSIG3_*IDC*_ (r = 0.66, p < 0.0001), VISTA_*TIIC*_ and VSIG3_*TIIC*_ (r = 0.43, p < 0.0001), VISTA_*TIIC*_ and CD45 (r = 0.31, p < 0.0001), VSIG3_*IDC*_ and VSIG3_*TIIC*_ (r = 0.61, p < 0.0001), VSIG3_*IDC*_ and CD45 (r = 0.33, p < 0.0001), VSIG3_*IDC*_ and CD68 (r = 0.29, p < 0.0001), PSGL-1_*IDC*_ and PSGL-1_*TIIC*_ (r = 0.36, p < 0.0001), PSGL-1_*IDC*_ and CD45 (r = 0.32, p < 0.0001), and PSGL-1_*IDC*_ and CD68 (r = 0.27, p < 0.0001), as showed in Fig. 3. Additionally, a statistical analysis showed a positive correlation between ER status and VISTA_*IDC*_ (r = 0.23, p = 0.0071).

**Fig. 4:**
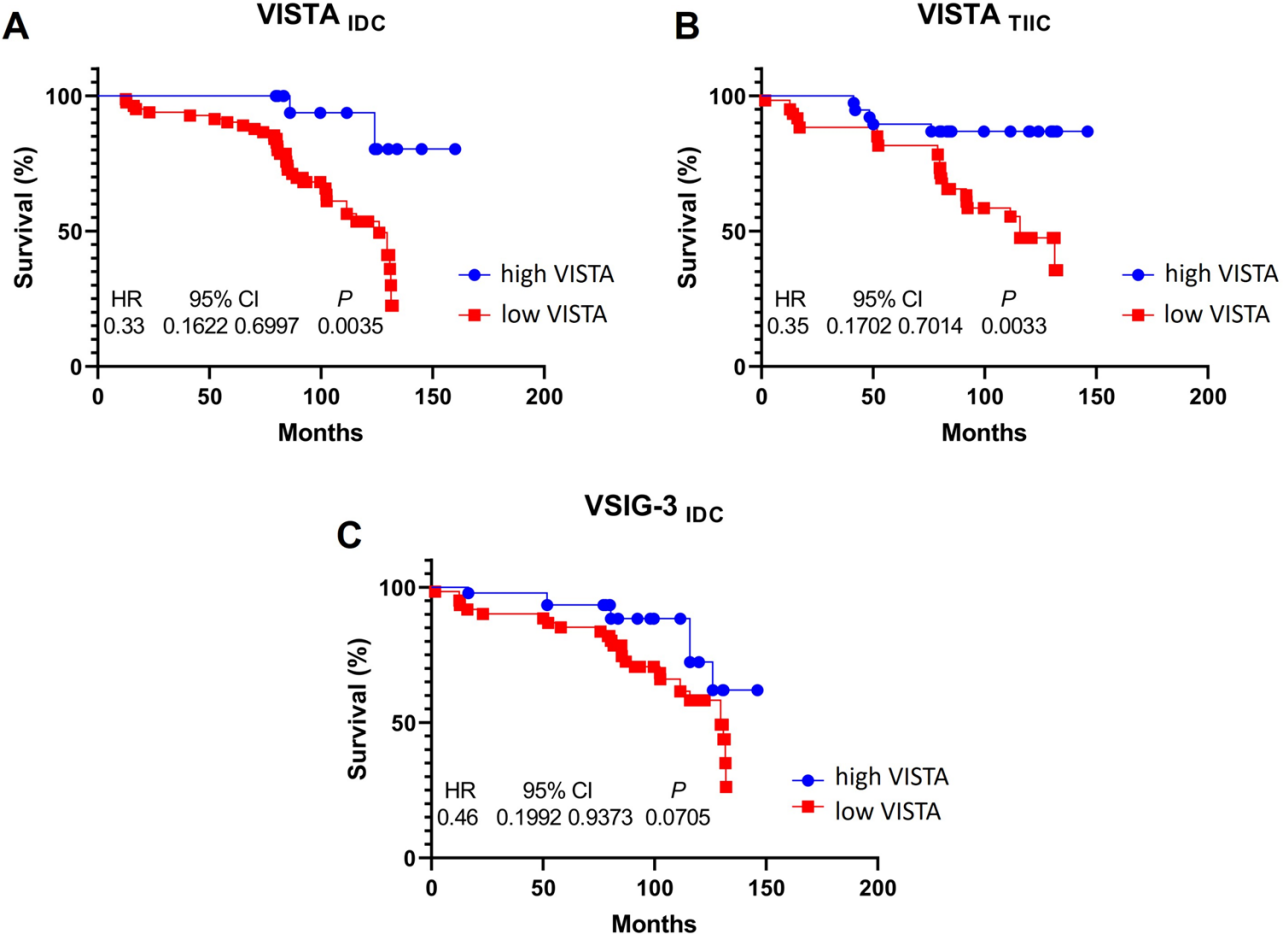
Survival time of IDC patients with regard to VISTA expression profiles. **A** The survival time of patients with a high expression of VISTA_IDC_ is higher than that of patients with a low expression of the VISTA_IDC_ protein (p = 0.0035). High VISTA_TIIC_ expression shows a better outcome on patients’ overall survival compared to low VISTA_TIIC_ expression levels (p = 0.0033) (**B**). **C** High levels of VSIG3_IDC_ protein expression are positively correlated with longer patient lifetimes (p = 0.0705) compared to patients with low expression levels

We used Kaplan–Meier to analyze the effect of the VISTA/VSIG3/PSGL-1 axis on the survival time of patients with IDC. We observed that higher overall VISTA expression, both in IDC and TIIC areas, had a positive impact on overall time compared to the groups with lower VISTA expression (VISTA_*IDC*_ p = 0.0035, HR = 0.33, 95% CI = 0.1622–0.6997; VISTA_*TIIC*_ p = 0.0033, HR = 0.35, 95% CI = 0.1702–0.7014) (Fig. 4A, B).

A similar situation was noticed for VSIG3 expression in IDC samples, whereas elevated VSIG3 expression levels correlated with a better overall survival time for IDC patients (p = 0.0705, HR = 0.46, 95% CI 0.1992–0.9373) (Fig. 4C).

Moreover, CD45-positive regions of tumor-infiltrating lymphocytes (TILs) were identified in 125 (96.89%) samples, and 128 (99.22%) cases showed CD68-positive regions of tumor-associated macrophages (TAMs). The expression levels of CD45 and CD68, ranging from low to high, were observed in only 16 (12.40%) and 12 (09.30%) BC cases, respectively. Moreover, the Spearman correlation test revealed a strong positive correlation between hormone receptor status and the tumor features of BC cells and the CD45- and CD68-positive regions. CD45 correlations with CD68 (r = 0.84, p < 0.0001), ER (r = 0.40, p < 0.0001), PR (r = 0.34, p < 0.0001), HER2 (r = 0.34, p < 0.0001), and pN (r = 0.29, p < 0.001), but not pT (r = 0.10), were noticed. Similarly, CD68 correlated with ER (r = 0.33, p0.001), PR (r = 0.33, p0.001), HER2 (r = 0.30, p0.001), and pN (r = 0.27, p0.001), but not with pT (r = 0.08).

### VISTA, VISG3, and PSGL-1 expression patterns vary among breast cancer cells

**Fig. 5:**
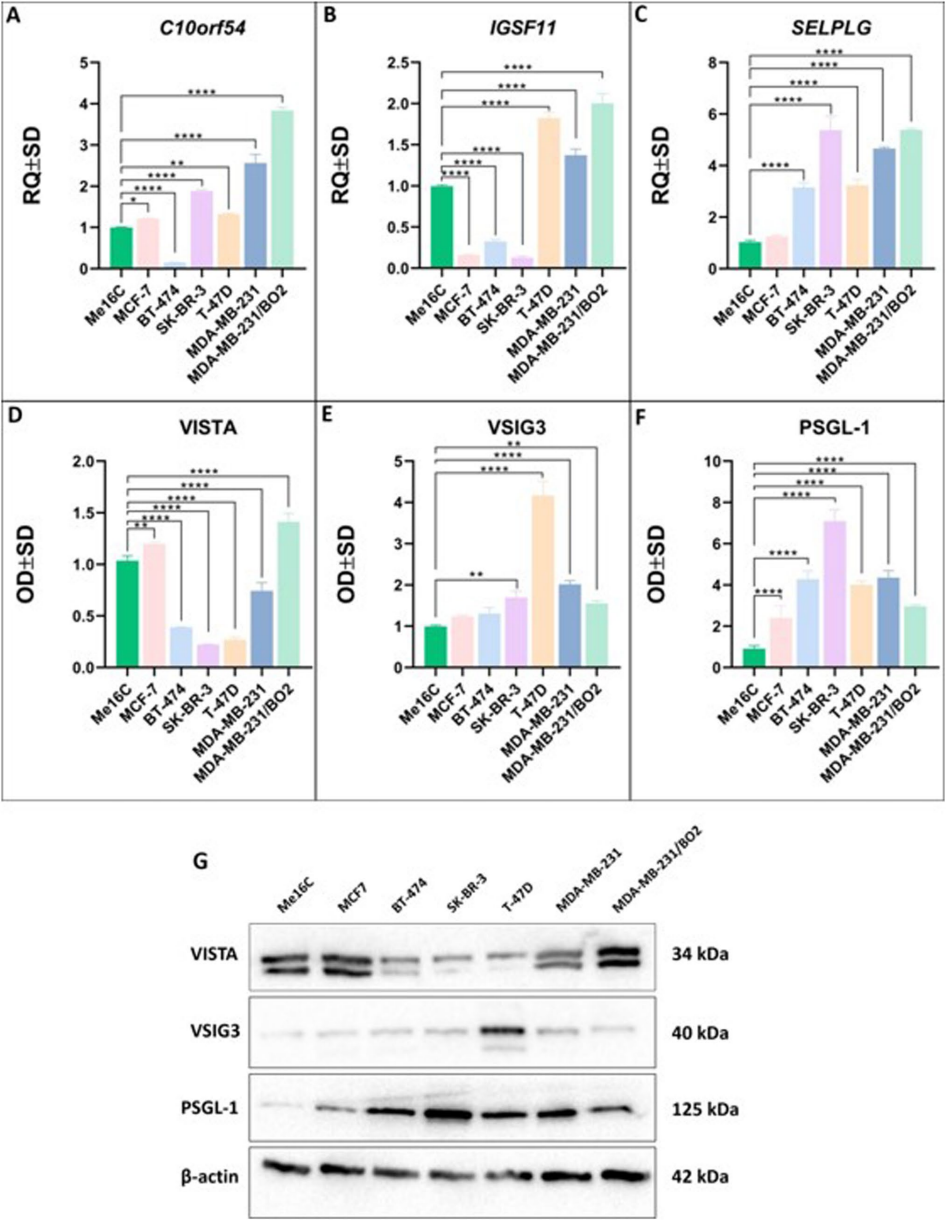
Relative levels of VISTA, VSIG3, and PSGL-1 mRNAs in MDA-MB-231 and T-D7D cells transfected with different siRNAs. Cells were transfected with the siRNAs for VISTA, VSIG3, and PSGL-1 and incubated for 24 h and 48 h. Relative expression levels (RQs) of VISTA/VSIG3/PSGL-1 axis mRNAs after silencing of VISTA (**A**, **D**), VSIG3 (**B**, **E**), and PSGL-1 (**C**, **F**). The data represent the means and standard deviation of three independent experiments. Comparisons between groups were conducted using the t test: *, p < 0.1; **, p < 0.01; ***, p < 0.001; ****, p < 0.0001

To evaluate the functional role of the VIST/VSIG3/PSGL-1 receptor pathway, its expression levels were examined in different human breast cancer cell lines, including MCF-7, BT-474, SK-BR-3, T-47D, MDA-MB-231, MDA-MB-231/BO2, and Me16C, as normal epithelial cell lines, as shown in Fig. 5. The increased aggressiveness of cancer cells leads to a significant increase in the expression levels of the studied transcripts and their protein products. These results indicate that the most elevated gene expression levels were present in the most aggressive cell lines, T-47D (*C10orf54* p < 0.01, *IGSF11* p < 0.0001, *SELPLG* p < 0.0001) and MDA-MB-231 (*C10orf54* p < 0.0001, *IGSF11* p < 0.0001, *SELPLG* p < 0.0001), compared to Me16C cells (Fig. 5).

Therefore, the T-47D and MDA-MB-231 cell lines were selected as optimal cell models for in vitro siRNA knockdown experiments. Protein level analysis using the WB technique may support the statement that the elevated expression of the VISTA, VSIG3, and PSGL-1 receptors increases with the aggressiveness of breast cancer cells. VSIG3 and PSGL-1 were visibly overexpressed in the T-47D (p < 0.0001, p < 0.0001) and MDA-MB-231 (p < 0.0001, p < 0.0001) cell lines compared to the Me16C epithelial cell line (Fig. 5E–F). A similar trend in the rising expression levels of VISTA protein was observed in the most aggressive breast cancer cell lines (Fig. 5D).

### siRNA-related knockdown shows crosstalk in VISTA/VSIG3/PSGL-1 axis

**Fig. 6:**
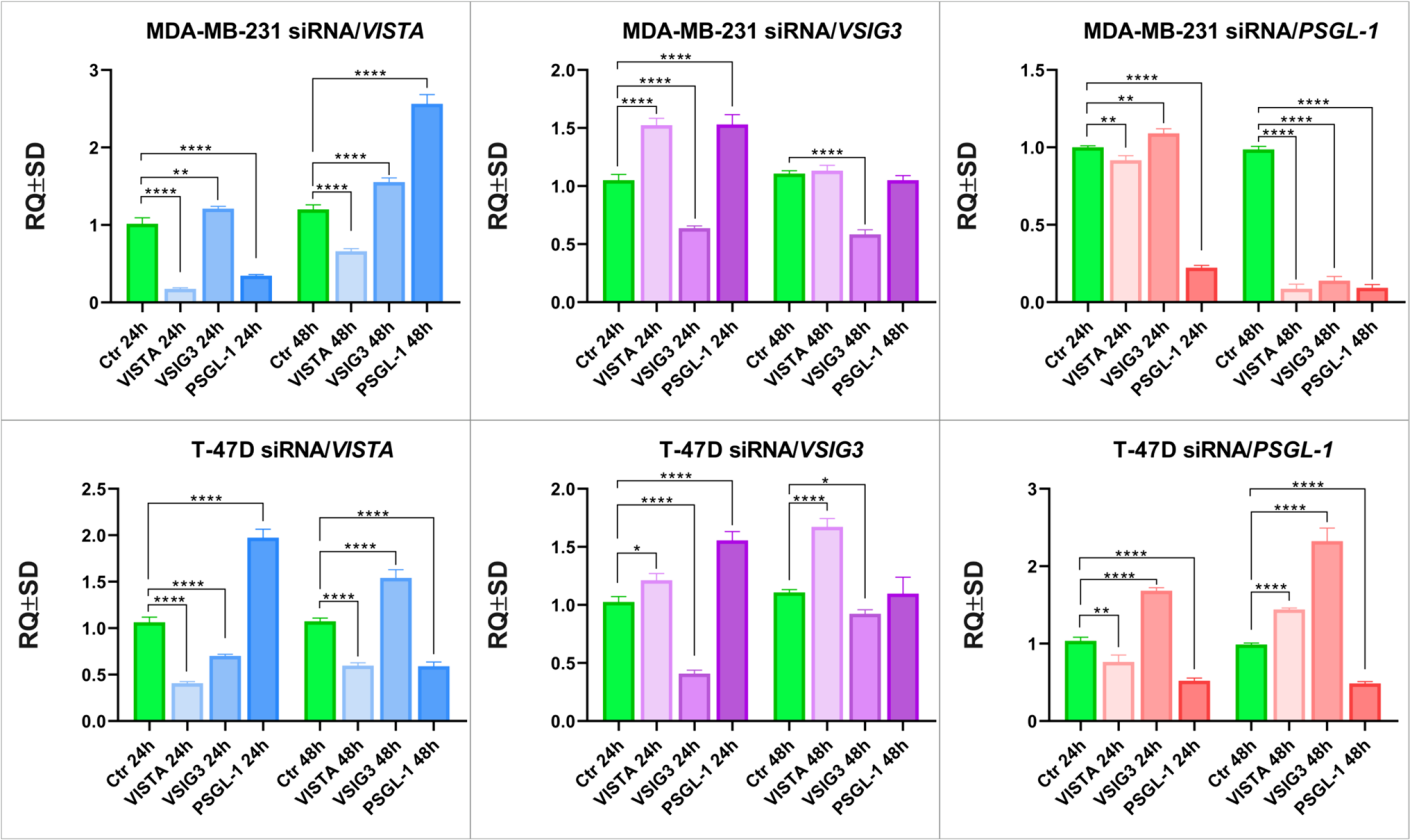
Relative levels of VISTA, VSIG3, and PSGL-1 mRNAs in MDA-MB-231 and T-D7D cells transfected with different siRNAs. Cells were transfected with the siRNAs for VISTA, VSIG3, and PSGL-1 and incubated for 24 h and 48 h. Relative expression levels (RQs) of VISTA/VSIG3/PSGL-1 axis mRNAs after silencing of VISTA (**A**, **D**), VSIG3 (**B**, **E**), and PSGL-1 (**C**, **F**). The data represent the means and standard deviation of three independent experiments. Comparisons between groups were conducted using the t test: *, p < 0.1; **, p < 0.01; ***, p < 0.001; ****, p < 0.0001

To evaluate the effect of siRNA-mediated silencing on the genes of the VISTA/VSIG3/PSGL-1 axis, specific siRNAs were transfected into the MDA-MB-231 and T-47D breast cancer cell lines for 24 h and 48 h. The efficacy of this transfection was measured using RT-qPCR and Western blotting. As shown in Fig. 6, we observed a statistically significant downregulation of all the silenced genes during the 24 h and 48 h time periods.

The downregulation of the VISTA gene in MDA-MB-231 cells resulted in overexpression of VSIG-3 after 24 h and 48 h (p < 0.001 and p < 0.0001, respectively) and a higher expression of PSGL-1 after 48 h (p < 0.0001) compared to the control cells. At the same time, in T-47D cells, overexpression of PSGL-1 after 24 h (p < 0.0001) and VSIG3 (p < 0.0001) after 48 h was observed. However, overexpression of VSIG3 after 24 h (p < 0.0001) and PSGL-1 after 48 h (p < 0.0001) was not observed. The downregulation of VSIG3 resulted in the overexpression of VISTA and PSGL-1 after 24 h in MDA-MB-231 cells. Similarly, in T-47D cells, we observed overexpression of VISTA (p < 0.1) and PSGL-1 (p < 0.0001) mRNAs after 24 h and overexpression of only VISTA (p < 0.0001) after 48 h. siRNA specific for the PSGL-1 transcript resulted in a significant reduction in the expression levels of VISTA (p < 0.01) and overexpression of VSIG3 (p < 0.0001) after 24 h. After 48 h, overexpression of both VISTA (p < 0.0001) and VSIG3 (p < 0.0001) was observed.

**Fig. 7:**
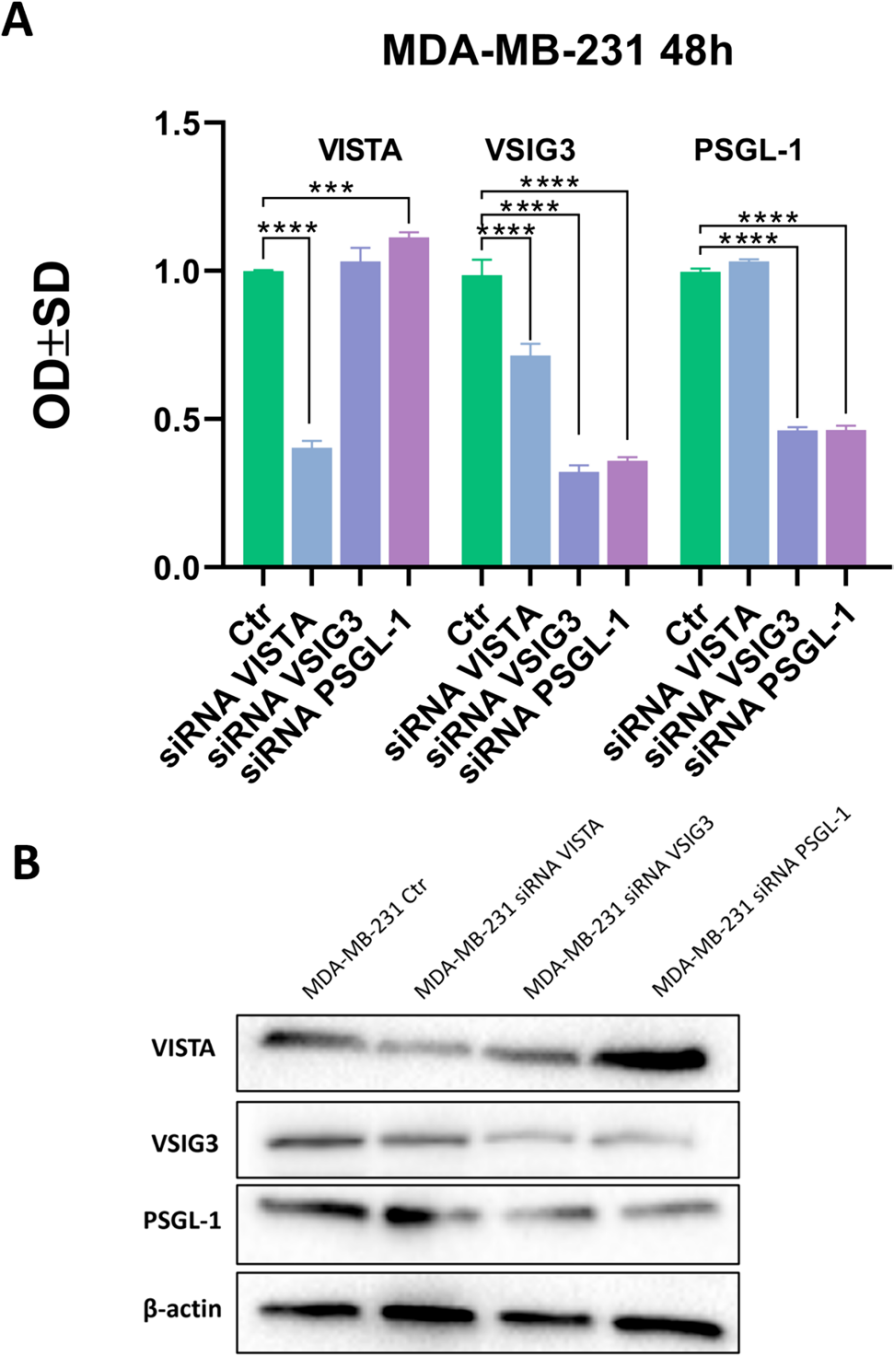
Western blot densitometric analysis of efficiencies of siRNA knockdown of VISTA, VSIG3, and PSGL-1 expressions in transfected MDA-MB-231 cell line after 48 h (**A**). **B** β-actin was used as an internal control. The data show the average standard deviation of three independent experiments. ***, p < 0.001; **** p < 0.0001. The results were identical after three repetitions of this experiment

Figure 7 illustrates that mRNA expression levels correlated with protein abundance in the MDA-MB-231 cell line after 48 h of gene knockdown. We observed statistically significant overexpression of PSGL-1 (p < 0.001) protein after VISTA knockdown, significant downregulation of VISTA (p < 0.0001) and PSGL-1 (p < 0.0001) expression after VSIG3 silencing, and a decrease in VSIG3 (p < 0.0001) after PSGL-1 inhibition.

## Discussion

The VISTA, VSIG-3, and PSGL-1 proteins are responsible for the regulation of the immune system and immune cells, as has been well documented. A recent study found that the binding interactions between VISTA and PSGL-1 are pH-dependent, and VISTA was identified as a binding partner for VSIG3 (also known as IGSF11) in two independent protein interaction studies [21, 23, 64]. To date, VISTA, VSIG-3, and PSGL-1 have not shown any well-established direct interactions or known relationships in the context of cancer. In recent years, the direct binding capacity of these proteins has been demonstrated in mouse antibody models using an SG7 inhibitor [65]. To our knowledge, this is the first study demonstrating the significance of the VISTA/VSIG3/PSGL-1 axis in human cancer cells. According to our findings, VISTA/VSIG3/SPGL-1 proteins are expressed in breast cancer cells as well as in lymphocytes (CD45 +) and macrophages (CD68 +), both of which are immune cells that infiltrate tumors. An increase in breast cancer cell aggressiveness correlates with an increase in the levels of proteins within the VISTA/VSIG3/PSGL-1 axis, as we have observed. These receptor levels are simultaneously overexpressed both in the most aggressive types of BC and on the surfaces of immune system cells. Notably, stromal expression of the VISTA receptor was observed in nearly 10% of the studied IDC cases. The importance of this discovery has yet to be described because of the lack of sufficient data [66–68]. Nevertheless, further studies are needed to determine the role of the VISTA/VSIG3/PSGL-1 axis in the tumor microenvironment and BC progression. The specificity of the IHC reaction shows that only very adjacent stromal cells are VISTA-positive, which may implicate their role in crosstalk between cancer cells, tumor-infiltrating immune cells (TAMs and TILs), and the extracellular matrix.

Results from both our own and previously published research on human breast cancer indicate that poorly differentiated BC cells have higher rates of VISTA expression. Since poorly differentiated tumors frequently contain cancer stem cells, it is reasonable to report that high VISTA expression in patients with high proportions of cancer stem cells likely serves as a mechanism of immune evasion and resistance to immunotherapy. Cancer stem cells are self-renewing cells with a high potential for tumorigenicity that reside in specific tumor microenvironment niches. Within this microenvironment, cancer stem cells are thought to be one of the main causes of immunosuppression. Few studies have been conducted in this field. Nevertheless, one important molecular explanation for the detrimental effect of immune checkpoint on cancer immunotherapy is the increase in the percentage of cancer stem cells and their interaction with cells inside the tumor’s immune microenvironment and inflammatory infiltrate. Immune checkpoint protein targeting may be viewed as an innovative tactic that enhances standard immune checkpoint therapy in reviving the antitumor side of tumor immunity, decreasing tumor recurrence, and producing long-lasting effects.

These findings highlight the significance of tumor stroma and microenvironment remodeling in cancer progression. The effective manipulation of all stromal components, for example, the extracellular matrix, fibroblasts, endothelial cells, and immune system cells, remains at the root of successful cancer evolution. Many components, such as innate and adaptive immune cells, play many functions during cancer progression and can either promote or suppress tumor formation, depending on local and systemic factors. Our findings strongly support the assertion that the expression of VISTA/VSIG3/PSGL-1 in both cancerous and immune cells may play a key role in the interactions between BC cells and their microenvironment. Changes in the expression levels of these receptor genes following siRNA knockdown further illustrate how easily and freely cancer cells may modify their gene expression processes to help them survive in their niche. This could be due to the fact that VISTA is a co-inhibitory molecule that lowers T-cell-mediated immunity while promoting immune escape. Our findings support the relevance of the VISTA/VSIG3/PSGL-1 axis as a biological target for immunotherapy and a predictive biomarker in breast cancer. Interrelationships and linkages between VISTA/VSIG3/PSGL-1 were observed at the RNA and protein levels at the same time.

VISTA is an immunological checkpoint protein, and its overexpression, together with increased levels of the VSIG3 and PSGL-1 receptors in the tumor microenvironment, may be involved in the modulation of immune responses to cancer. There is growing evidence of the importance of the immunosuppressive role of VISTA in cancer progression [20, 41, 57, 62, 63, 69–73]. The presence of VISTA on the surface of cancer cells can not only modulate immune cells but also potentially play a much broader role in cancer biology. Targeting multiple immune checkpoints may be a turning point in cancer therapy. Therefore, in this study, we investigated the crosstalk between VISTA and its receptors, VSIG3 and PSGL-1. Our research revealed a strong correlation between VISTA expression and PSGL-1 and VSIG-3 in IDC samples, indicating that this axis functions in concert with immune cells to promote the advancement of BC. In cases of breast cancer, inhibiting the VISTA pathway could improve antitumor immunity. When combined with other treatments, such as chemotherapy and radiotherapy, inhibition of the VISTA pathway could effectively limit tumor growth and reduce inflammation, which is helpful in the management of autoimmune disorders.

Nevertheless, further investigation of the role of VISTA in breast cancer and its potential as a therapeutic target may provide useful insights into the development of novel treatment options for this disease.

## Conclusions

The current study has demonstrated that the expression patterns of the VISTA/VSIG3/PSGL-1 axis were increased in infiltrating breast cancer cells and immune cells. To the best of our knowledge, this is the first study to show direct crosstalk between these immunological receptors, resulting in greater adaptation of tumor cells to their microenvironment. The knockdown of one of the receptors by siRNA resulted in an increase in other investigated genes, indicating the relevance of the VISTA/VSIG3/PSGL-1 axis in breast cancer progression. Despite our important findings, there remains a significant amount of work yet to be accomplished in this field to demonstrate the entire molecular basis of tumor microenvironmental mechanisms, as well as the role of VISTA receptors and their partners in immune regulation and cell interactions in human malignancies.

## Data Availability

All data associated with this study are present in the manuscript. Materials used in this study are available from the corresponding author upon reasonable request.
